# Supplementary material for: A Blood Biomarker for Duchenne Muscular Dystrophy Shows That Oxidation State of Albumin Correlates with Protein Oxidation and Damage in Mdx Muscle
Source: Antioxidants (Basel). 2021 Aug 3;10(8):1241. doi: 10.3390/antiox10081241 (PMC8389308; doi:10.3390/antiox10081241)
Supplement: Supplementary file 1 [file antioxidants-10-01241-s001.zip › antioxidants-1229440-supplementary.pdf]

Supporting information

A blood biomarker for Duchenne muscular dystrophy shows that oxidation state of albumin correlates with protein oxidation and damage in mdx muscle

Basma A. Al-Mshhdani[1], Miranda D. Grounds[2], Peter G. Arthur[1] and Jessica R. Terrill[1].

[1]School of Molecular Sciences, and `School of Human Sciences, the University of Western Australia, 35 Stirling Highway, Perth Western Australia.

Corresponding Author

Jessica Terrill

School of Molecular Sciences M310

University of Western Australia

35 Stirling Highway, Crawley

Western Australia, 6009

Phone: + 61 8 6488 1211

jessica.terrill@uwa.edu.au

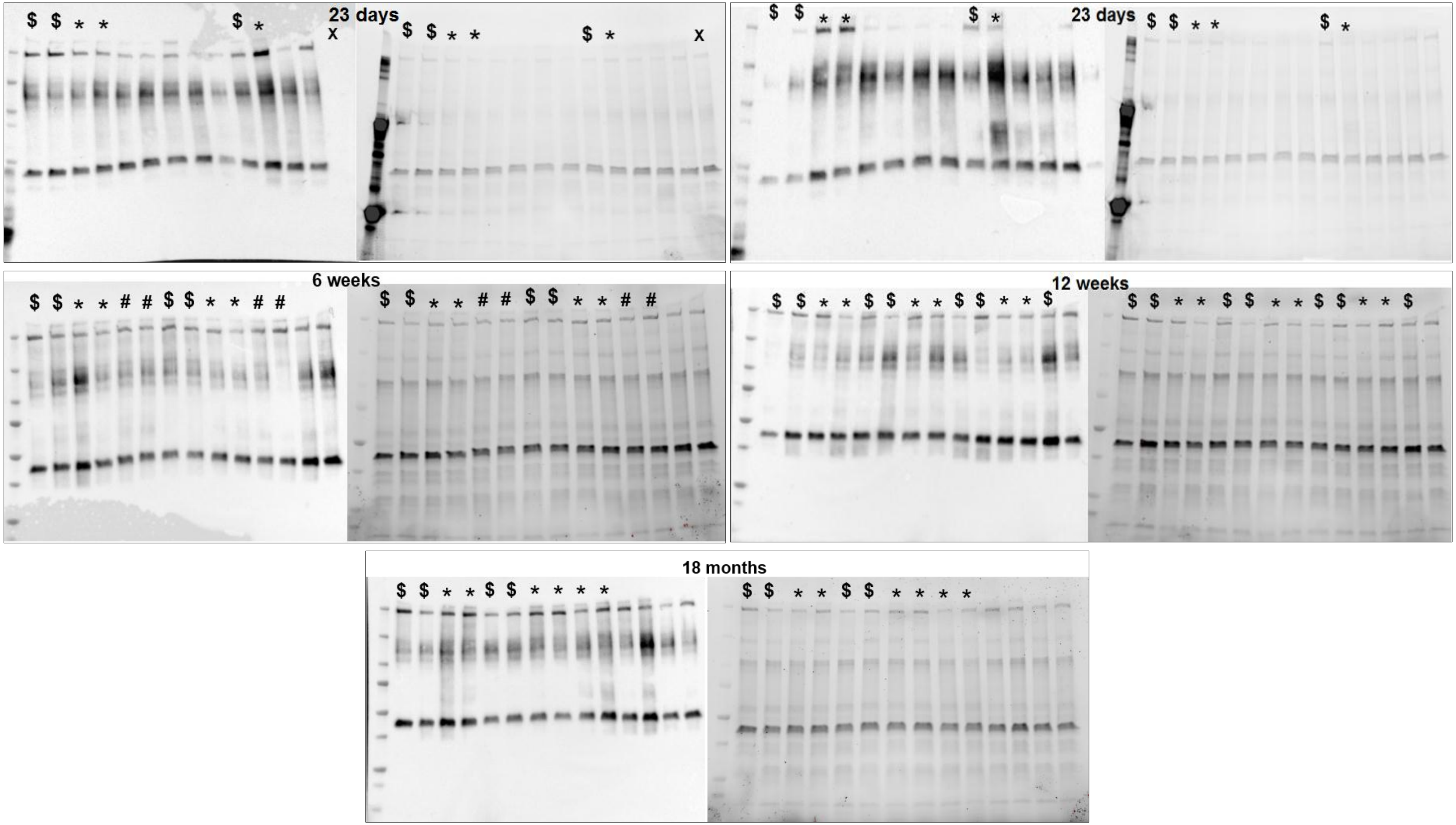

Muscle carbonyl (Fig 2 B): Healthy wildtype controls = $, Dystrophic animals (*mdx*) = *, *mdx*+ exercise= #, left side of the images represent western blot image (Immunoblot image), right side of the images represent gel image.

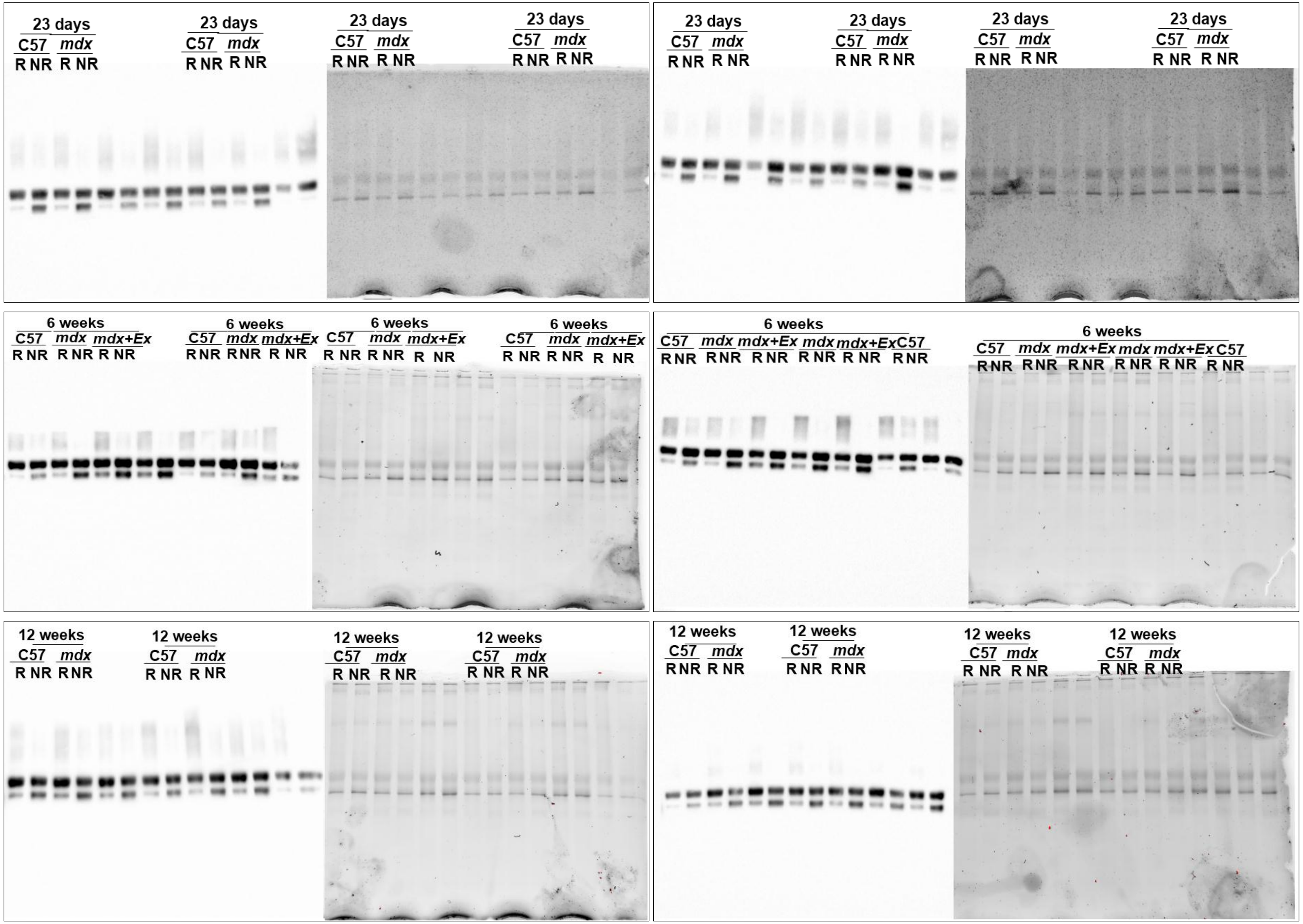

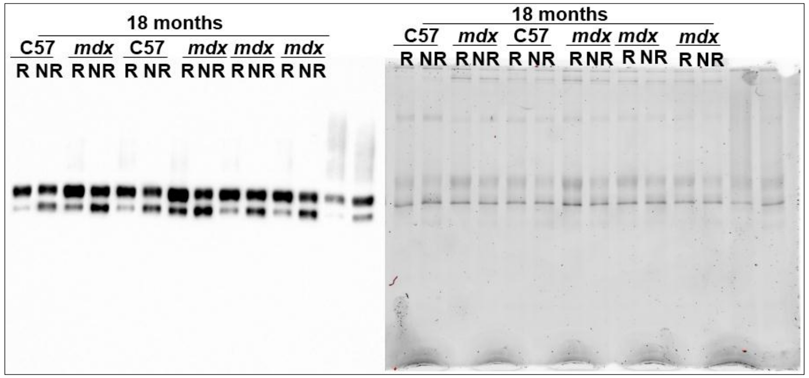

Plasma albumin thiol oxidation (Fig 3 A, B, and C).

R= reduced, NR= non-reduced sample,

mdx+exercise= *mdx*+ *Ex*.

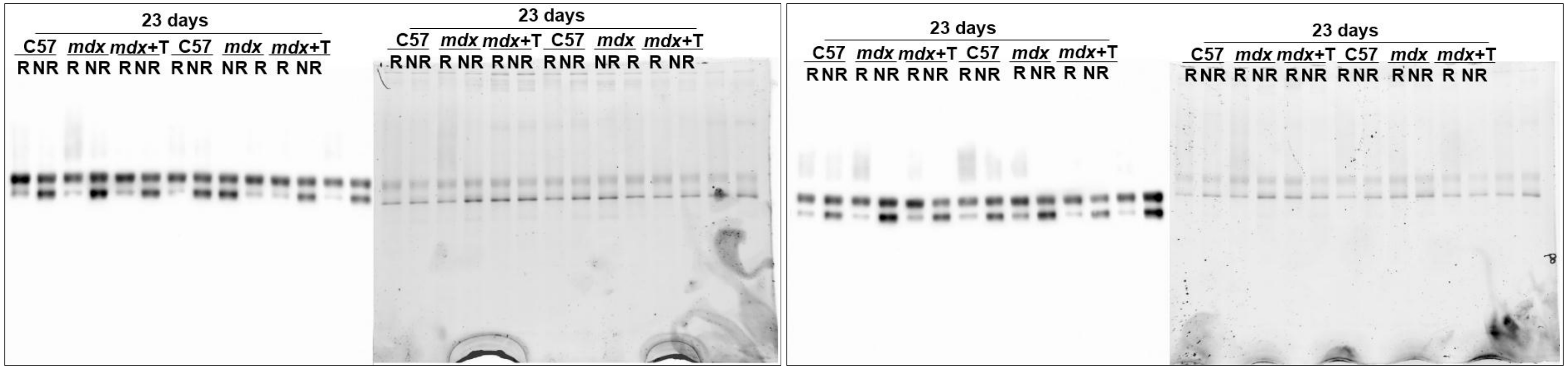

Plasma albumin thiol oxidation (Fig 5 C, D, and E).

R= reduced, NR= non-reduced sample, *mdx*+T= *mdx*+ taurine.

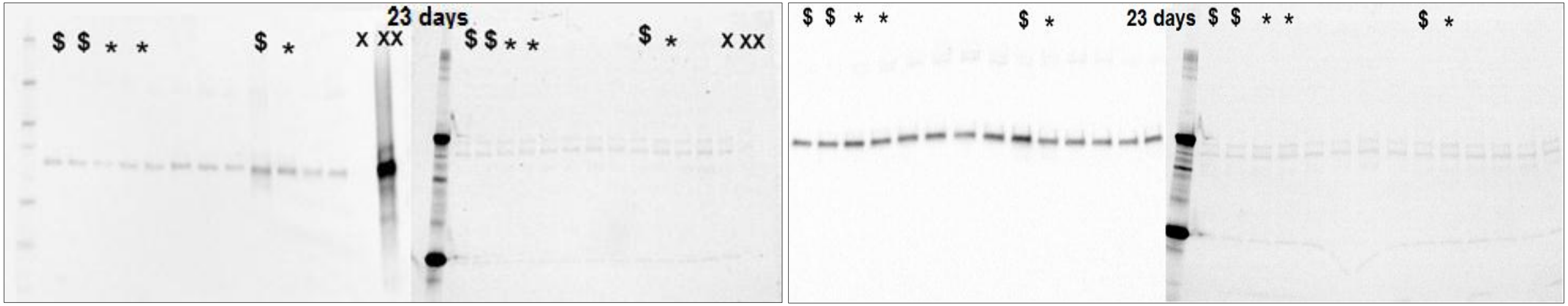

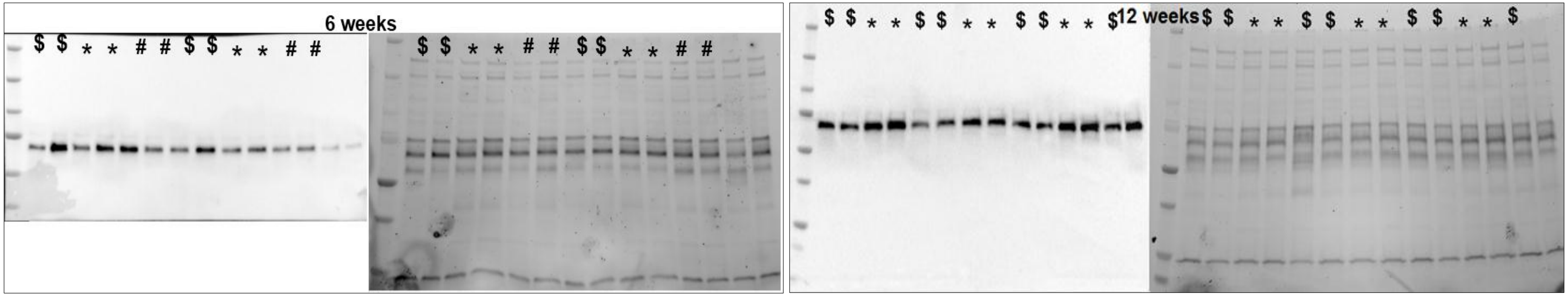

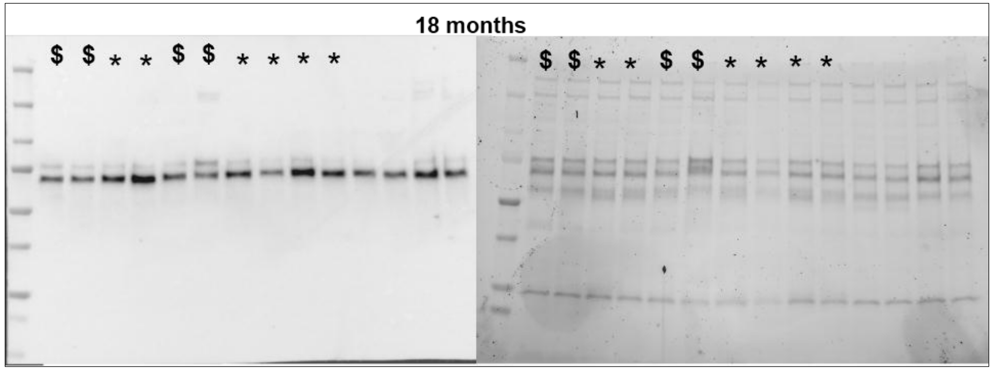

Plasma protein carbonyl (Fig 3 D).

Healthy wildtype controls = $, Dystrophic animals (*mdx*) = *, *mdx*+exercise= #, x= plasma sample without labelling with DNPH for use as a negative control, xx= a plasma sample from a C57 mouse was incubated with 1 mM HOCL prior to DNPH labelling for use as a positive control.
